# Smart Toys Designed for Detecting Developmental Delays

**DOI:** 10.3390/s16111953

**Published:** 2016-11-20

**Authors:** Diego Rivera, Antonio García, Bernardo Alarcos, Juan R. Velasco, José Eugenio Ortega, Isaías Martínez-Yelmo

**Affiliations:** 1 Departamento de Automática, Escuela Politécnica Superior, Universidad de Alcalá. Campus Universitario, 28871 Alcalá de Henares, Madrid, Spain; diego.rivera@uah.es (D.R.); antonio.garciah@uah.es (A.G.); bernardo.alarcos@uah.es (B.A.); isaias.martinezy@uah.es (I.M.-Y.); 2 Facultad de Psicología, Departamento de Psicología Biológica y de la Salud, Universidad Autónoma de Madrid, Calle Iván Pavlov, 6, 28049 Madrid, Spain; eugenio.ortega@uam.es

**Keywords:** Internet of Things, intelligent toys, health, children

## Abstract

In this paper, we describe the design considerations and implementation of a smart toy system, a technology for supporting the automatic recording and analysis for detecting developmental delays recognition when children play using the smart toy. To achieve this goal, we take advantage of the current commercial sensor features (reliability, low consumption, easy integration, etc.) to develop a series of sensor-based low-cost devices. Specifically, our prototype system consists of a tower of cubes augmented with wireless sensing capabilities and a mobile computing platform that collect the information sent from the cubes allowing the later analysis by childhood development professionals in order to verify a normal behaviour or to detect a potential disorder. This paper presents the requirements of the toy and discusses our choices in toy design, technology used, selected sensors, process to gather data from the sensors and generate information that will help in the decision-making and communication of the information to the collector system. In addition, we also describe the play activities the system supports.

## 1. Introduction

It is clear that children use toys everywhere. We can think that toys are their main interface with the world. In this sense, thinking about games or toys as interfaces in order to detect, as soon as possible, developmental difficulties or even disorders in children, becomes natural. Childhood professionals develop activities with children that manipulate classical toys, in order to evaluate their psychological and physical evolution. Thus far, an analysis of children’s interaction with interesting objects in different periods of development, led to propose the use of toys as rattles, balls and/or cube towers in order to study children’s motor skills and development between the ages of 0 and five years [1,2].

There are some developmental scales, such as Merrill–Palmer [3], that allow the identification of developmental delays and learning difficulties. These scales provide some activities using toys, and define goals that children of different ages must reach properly. The examiner must verify if the child is able to do it successfully within a predefined time.

As said before, toys used in the developmental scales use to be conventional objects, like cube towers or rattles. The examiner only obtains rough information from the activities to decide about the presence of a disorder.

By introducing sensors inside the toys, it will be possible to give the examiner more and better information to detect disorders in standard children. Consequently, the detection of developmental delays could improve their sensibility.

The goal of this research is to enrich the accuracy of traditional evaluation methods by embedding sensors into daily life toys that provide professionals with added value supplementary information enhanced by decision-support systems.

This paper presents the design of a smart toy with embedded sensors that gives new and interesting information to childhood development professionals. The toy and the kind of information have been agreed on by a working group of multidisciplinary researchers specialised in disciplines of childhood development and computer science.

The first toy selected by the working team is a set of cubes and the defined activity is to build a tower of cubes. The information that the development childhood researchers have identified as interesting are:

- Pattern of motion while the child is manipulating a cube.
- Time taken in build a tower of a number predefined of cubes.
- Accuracy in cubes alignment to build the tower.

**Figure 1:**
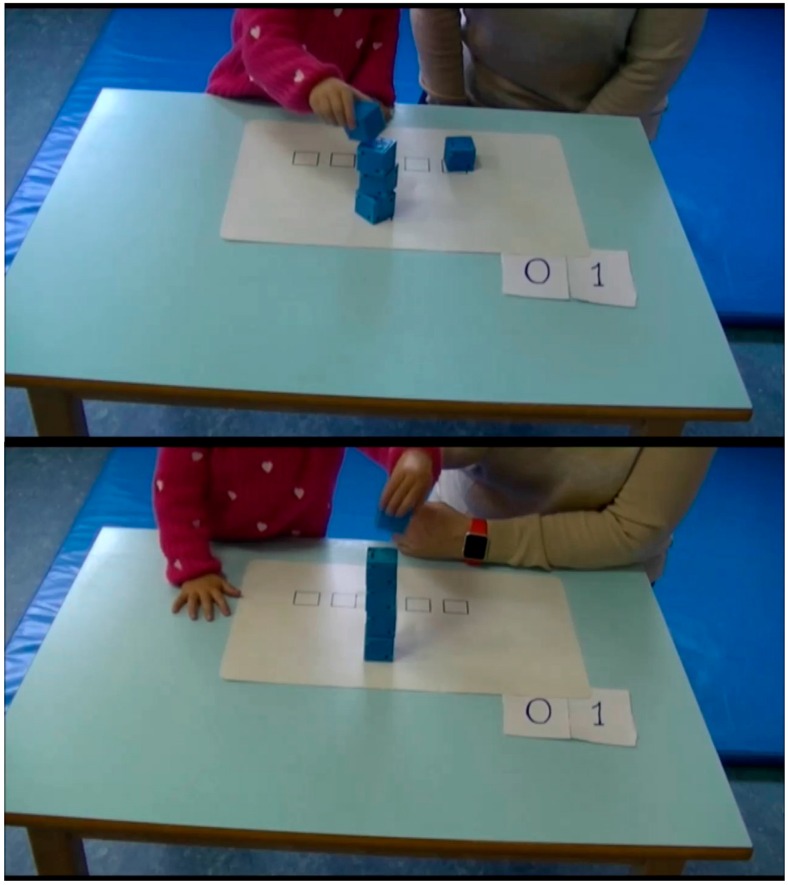
A child building a five-cube tower.

Figure 1 shows two different moments of a cube tower built by a child. It is known that in several children disorders, like Autism Spectrum Disorders (ASD), some information obtained from embedded sensors may be relevant for early diagnosis. In these cases, it has been proven that the movement is slower, the deceleration time is longer and the amplitude of speed peak is higher [4]. Moreover, maximum acceleration should be lower [5] and the time to reach the speed peak tends to be higher [6].

In fact, the idea of using games/toys in this area is not really new. In the frame of the “Games for Health” Project, a taxonomy (Table 1) of games is presented where several kind of games with different goals are described: self-ranking, Rehabilitation Disease management, data collection, etc. [7].

In this line, the EDUCERE Project [8], where the cubes we present in this paper are included, will develop different devices that may be embedded into toys or other common objects used at school, at home, or at the paediatrician office. These devices should capture user data, so teachers or doctors may detect outlier children from regular patterns.

The use of this kind of technology to detect behaviour patterns into the health field is becoming more common every day in what is known as wearable devices [9]. Marschollek provides a classification [10] of the different types of sensors that may be used, and analyse the possible cases of use. Albinali et al. present in [11] a group of children wearing three wireless accelerometers placed simultaneously on the left wrist, right wrist and on the torso, to detect stereotypical hand flapping and body rocking on individuals with ASD. A similar job is described in [12]. Although these kind of sensors are not intrusive strictly speaking, as children wear something that is not usual, it may alter their behaviour. This is the main reason to embed the sensors in common objects (mainly toys), so children are not affected.

Moreover, these toys might let us to automate some well-known tests like Denver or Merrill–Palmer, letting us to step forward what is described in [13], where a rule based system is used by paediatricians to detect developmental problems (in this case for the language field) in children.

In fact, the use of Internet of Things (IoT) has been identified as a valid technology to add value to disabled people, making the interaction with the physical word easier, avoiding the use of other objects but the common ones they use every day. Domingo [14] presents how these IoT devices avoid any normal life disturbance. The Internet of Toys (IoToy), as we name this flavour of the IoT, has been used in [15], but in a slightly different sense. In that work, the term IoToy was used to define electronic toys that are able to interact with the environment and the player by using his/her favourite social network.

Of course, these devices may be used to collect user data, but they might be used to motivate users to perform different activities too, as in [16]. In this study, an enhanced Frisbee-Disc as interaction device is presented. They use this device to explore the capability of flying tangible user interfaces for increasing the attractiveness of physical games. While playing with the disc, a dynamic audio stream is generated, depending on the quality of the flight. Some other examples may be found in [17] and [18]. In the first one, sensors are embedded in conventional objects, like a cushion that is used for rehabilitation therapy. In the second one, authors present the development of digital games for physical therapy with currently available commercial input devices.

The next section presents the architecture of the system based the cube tower toy, and the last section is devoted to results, conclusions and future work.

## 2. Description of the Smart Toys Architecture

### 2.1. General Description of the Architecture

The goal of the smart toys system is to provide the development professional enough information about the activity performed by children. In the use case presented in this paper, the activity is to build a tower of cubes. As we advanced in previous section, these professionals need to know the following parameters:

- Motion pattern while the child is moving a cube: time of activity, acceleration, speed, and shaking data.
- Tower status: They want to know if the children made the cube tower, how long did it take, how accurate was the alignment of cubes in the tower, etc.

**Figure 2:**
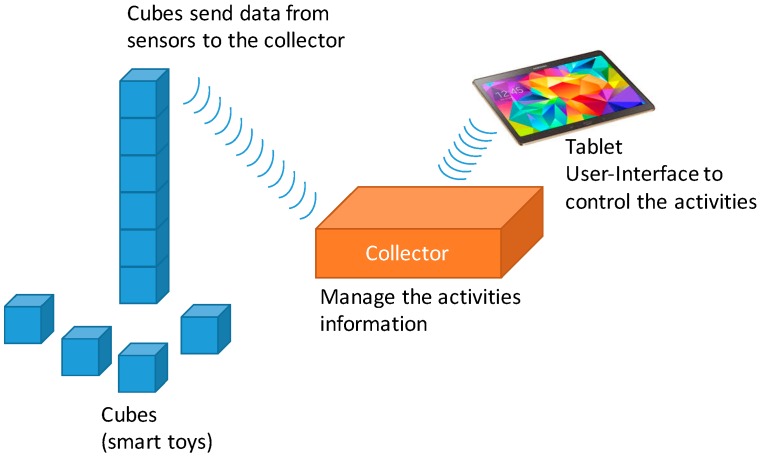
Physical architecture of the proposed smart toy.

To design the system, we need to select the appropriate kind of sensors to introduce in every cube, and send the information from the cubes to a data collector that stores it in a database. Figure 2 shows the physical architecture of the system.

The cubes send sensor data to the collector. When the activity has finished, the collector saves the received data from the cubes and the related data about the activity (date, time, person that manage the activity, child identifier, etc.).

In next sections, the different parts of this system are described in detail: the cubes design, the sensors used for motion detection, the sensors used for tower building detection and the collector system.

### 2.2. Cube Board

The cube design has some constrictions specified by the child development experts of the researching group. These specifications are:

- Dimension of the cube side between 2.5 and 3 cm.
- Easy to use for non-experts in computer science.
- Give information motion and quality of building of the activity.
- Enough autonomy to program working sessions of at least four hours without maintenance needs such as charging battery.
- Use low cost and easily accommodated technology to be used by do-it-yourself supporters.
- Easy reusability for other smart toys, like rattles.

The cube has a limited dimension and we need to introduce the following list of components:

- Microcontroller.
- Battery and associated protection circuits.
- Wireless communication system that sends sensor data to the collector.
- Sensors: accelerometer and Light-Dependent Resistor (LDR).
- Power Switch.
- User Interface: LEDs and buzzer.

We have integrated in the designs the user interface to give us the chance to program signals. For example, we may make a sound to indicate that some error has happened or to catch the attention of the child in the activity. A led can be switched on with the same purpose.

The selected processor to build the cube is the ATmega328p from ATMEL. We have selected this microcontroller because it has enough processing capacity to manage the sensors of a toy and is used by the open hardware technology based on Arduino which uses a well-known IDE for programing.

**Figure 3:**
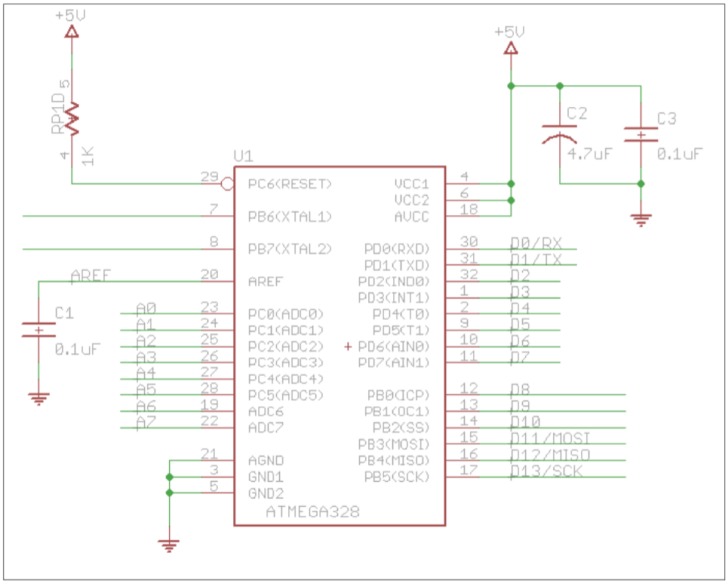
Microcontroller schematic.

Considering that the system needs to be powered by a battery and that the dimension of the cube is limited to 3 cm, the Arduino prototype board is not valid for this project because it is too big and furthermore has some unnecessary components that increase the energy consumption (USB chip, voltage regulator, etc.). Figure 3 shows the basic design of the microcontroller ATmega328P (Atmel Corporation (San Jose, CA, USA)) with minimum configuration.

Capacitors C1, C2 and C3 with the values recommended by the manufacturer and resistor R1 is the minimum configuration of the microcontroller. External clock has not been included in the circuit, instead we use the internal clock circuit of 8 MHz that is slower that the external of 16 MHz, but we consider that 8 MHz is enough for the proposal of the project, and as an added advantage consumption is lower.

As energy supply, a LiPo battery (Polymer Lithium Ion) of 3.7 V has been chosen because it has a good size/capacity ratio. The limitation of the cube size implies some limitations in the capacity of the battery. The largest side of the battery may have a maximum length of 2.5 cm. The largest capacity we have found with this length limitation is 150 mAh.

**Figure 4:**
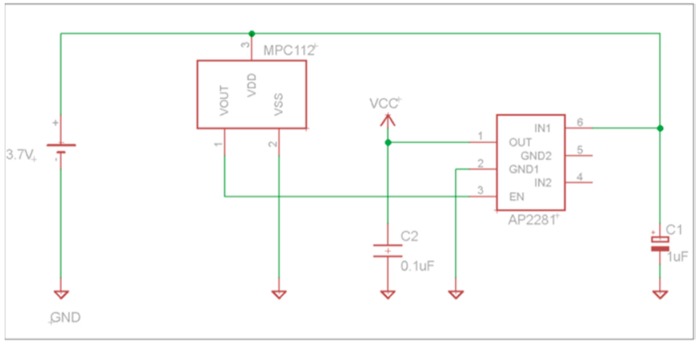
Schematic of protection discharge circuit.

One of the difficulties using LiPo batteries is that they should not be discharged below approximately 3.2 V, so an electronic protection circuit has been implemented in order to monitoring the discharge process and switch off the source when the battery reaches the threshold of 3.15 V. This circuit consists of a chip MCP112 (Microchip Technology Inc. (Chandler, AZ, USA)) (see Figure 4) that measure the voltage of the battery (pin 3 of MCP112) and the chip AP2281 (Diodes Incorporated (Plano, TX, USA)) that acts as switch, when the voltage in the battery is higher than 3.15 volts, the VOUT (pin 2 of MCP112) of the circuit enable the switch (AP2281) and the battery voltage IN1 (pin 6 of AP2281) pass to the OUT (pin 1 of AP2281) switching the supply voltage to the rest of the circuit.

One challenge of the cube design is switching off the cube when it is not being used. In order to avoid having to install a mechanic switch in the cube, a relay reed has been fitted. The relay switches off the cube circuit when a magnetic field has been applied. In this way, hidden Neodymium magnets are built into the box that keeps the cubes, so the cubes can be switched off when they are placed into the box. Furthermore, this box integrates the battery chargers, one for each cube. The charger is connected by a male connector plugged into two small holes in the cube.

The cubes have to send information processed from sensors to the collector system. To send this information, we need to select a wireless technology. The requirements are:

- Low consumption.
- Low cost.
- Easy to integrate in ATmega328 microcontrollers.
- Size less than 2.5 cm.

We have considered (see Table 2) different products working on the band of 2.4 GHz. A RF 315 MHz device has been analysed too, but it was not finally considered due to size constraints because of the use of two different elements as transceiver and receiver.

The three devices are easy to integrate with ATmega328 microcontrollers. For the NRF24L01+ (Nordic Semiconductor ASA (Oslo, Norway)) device there are software libraries available. In the case of the Bluetooth and WIFI devices there is the possibility to configure de device using AT commands via serial interface. The size of the Bluetooth interface is bigger than 2.5 cm so does not fulfil one of the requirements. Finally, the NRF24L01+ has been the selected radio frequency (RF) device because it is more energy-efficient than the WIFI device and fulfils the size requirements.

**Figure 5:**
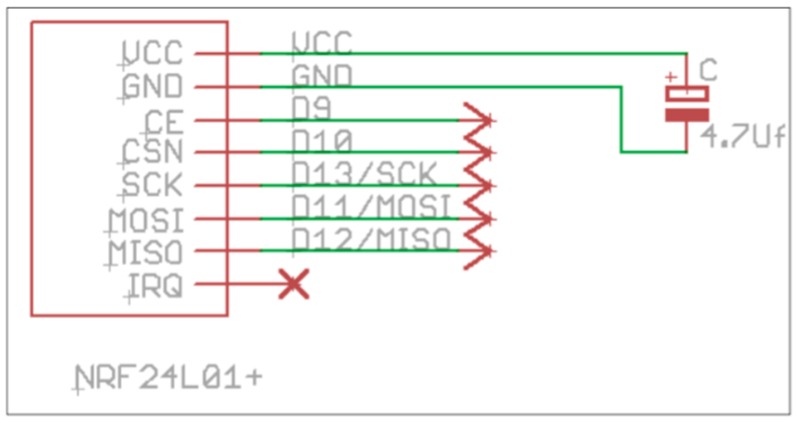
Communication technologies analysed.

The NRF24L01+ implements a link layer with automatic ACK and lost frames recovery. This is enough for our purposes. The maximum data rate provided by the device is 2 Mbps, but we will configure it to work at the default rate of 1 Mbps. We consider this data rate valid for the purpose of the project and also the consumption is lower. This RF device is connected to the microcontroller via Serial Peripheral Interface (SPI) (Figure 5).

The IRQ pin out can be connected to a pin interruption of the microcontroller. This possibility does not have been considered in this first version but we consider that could be an interesting possibility for future research if we conclude that should be necessary to save more energy.

**Figure 6:**
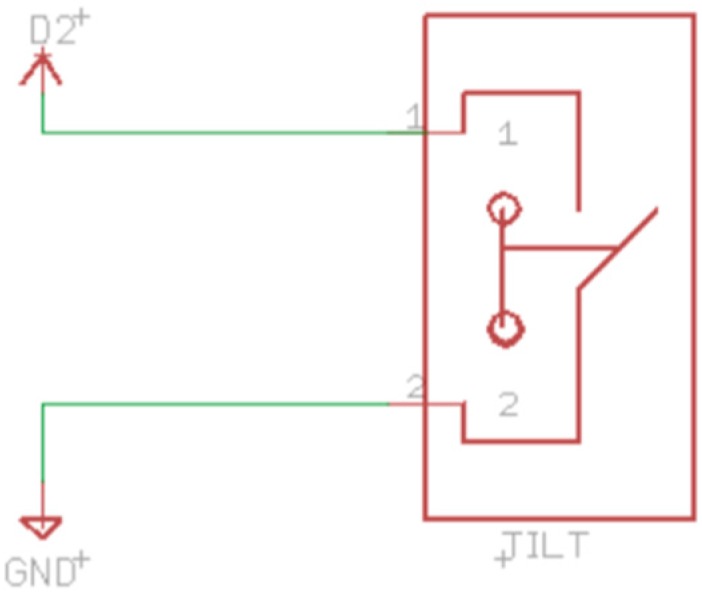
Tilt schematic.

In order to optimise the energy consumption, the microcontroller should be sleeping when there is no need of processing. In order to detect if the toy is being manipulated, we have installed a tilt sensor. A tilt is a passive sensor that detects orientation or inclination, acting as a switch. Thus, it can be used to detect motion of the toy. Figure 6 shows that the tilt is connected to the pin D2 of the microcontroller. Therefore, the microcontroller can wake up when the cube is being shaken.

**Figure 7:**
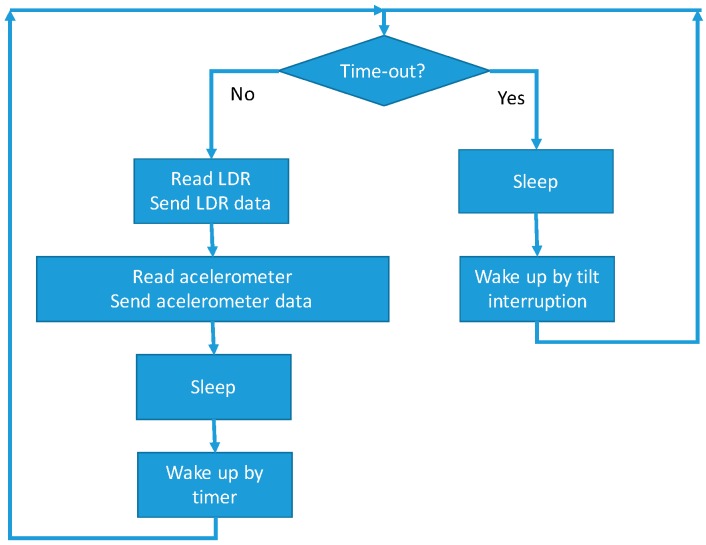
Logic for sleeping methods.

Another possibility that we have evaluated is to sleep the microcontroller and wake it up by internal timer. The configuration of both sleeping methods may be properly combined in order to sleep when there is no physical activity (sleep by expiry timer without activity and wake up by tilt interruption) and when the microcontroller does not have a task to do (sleep and wake up by expiry timer). In Figure 7, we can see the logic of both sleeping methods combined. This scheme has been developed in validation tests presented below.

The sleep method selected is SLEEP_MODE_PWR_DOWN because it is the method that saves the most energy.

**Figure 8:**
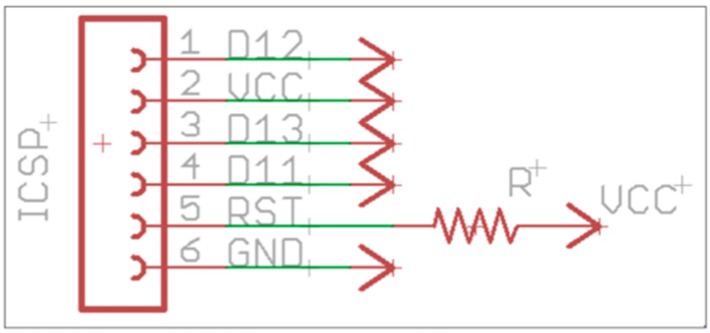
Programing interface.

The programing method proposed to send the developed programs to the microcontroller is via the ICSP interface. Figure 8 shows the ISCP pinout. Using this programing method implies the use of a programmer device, but it is not expensive and provides the following advantages: (1) memory saving in the microcontroller because is not necessary to install a bootloader; and (2) energy saving because it is not necessary to add the USB chip used to program the Arduino boards using bootloader.

The selected sensors to be included in the cube are:

- Accelerometer MPU-9150.
- LDR (Silonex Inc. (Montreal, QC, Canada)) light sensors to send information about the sides of the cube that receive light.

**Figure 9:**
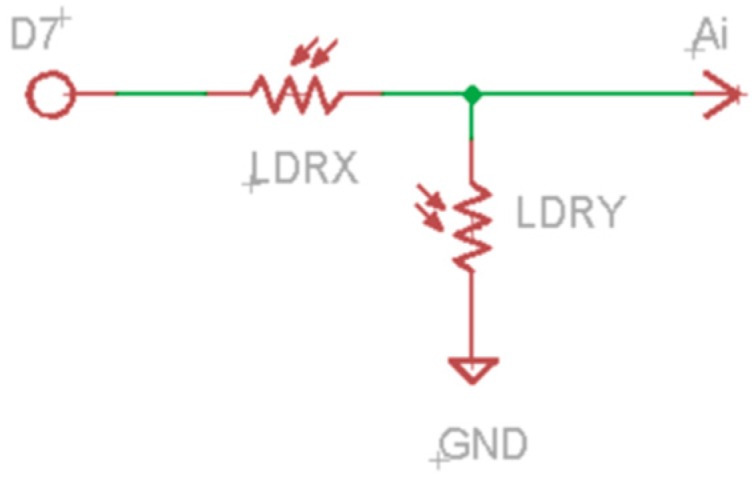
LDR schematic.

**Figure 10:**
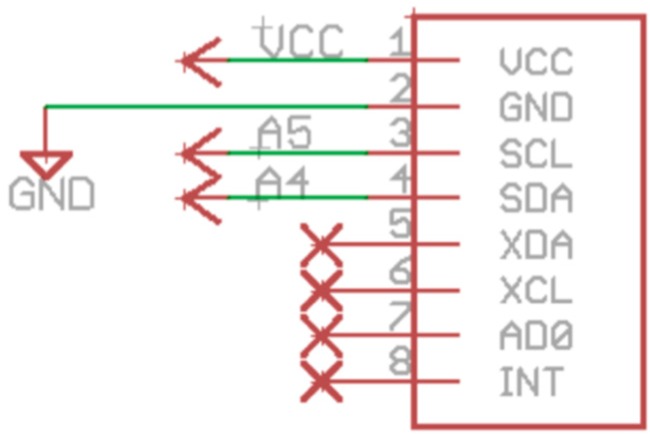
Accelerometer schematic.

The use of these two sensors is discussed in different sections below. Figure 9 and Figure 10 show the schematic to connect both kinds of sensors to the microcontroller.

LDRs are connected in series making a voltage divider. The divisor output is connected to the analogic inputs of the microcontroller. There is a LDR divisor for each face of the cube. The power source of the divisors is connected to the digital output D7 of the microcontroller. This way, D7 is usually low level and only goes high when measures must be taken. This is another technique used for energy saving.

The accelerometer is connected to the microcontroller via I2C interface as shown in Figure 10.

**Figure 11:**
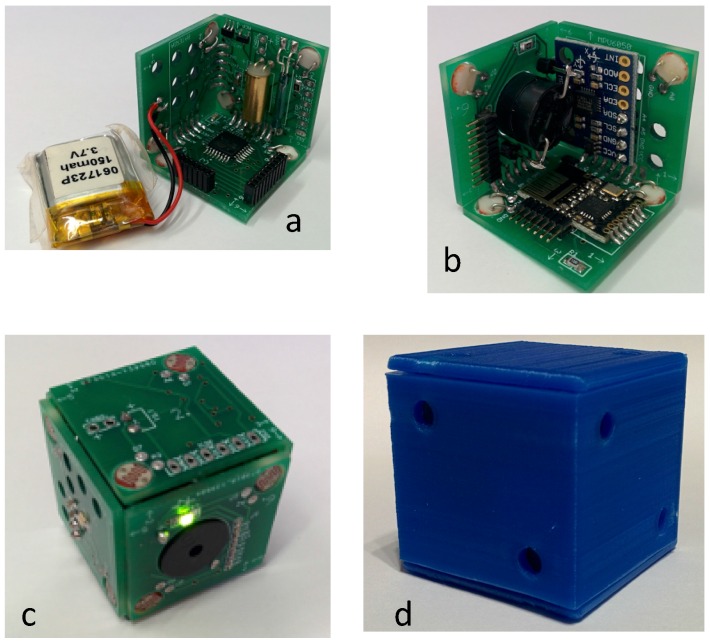
Picture of the cube. (a-b) two half-cube pieces; (c) assembling pieces; and (d) the cube with plastic housing.

The proposed shape for the printed circuit board (PCB) design is a cube. The PCB cube consists of two pieces joined by connectors. Each of these pieces represent three faces of a cube joined by two sides and a vertex. Each face of the cube is a 2.54 × 2.54 cm square PCB. The union of two faces forming an angle of 90° is done through welding paths that end at the edge of each joined face. All the electronic components apart from LEDs have been welded on the inside faces of the cube. Figure 11 shows cube pictures: (a, b) two half-cube pieces; (c) assembling pieces; and (d) the cube with plastic housing.

Some testing program have been developed to validate that the hardware is working properly:

- Accelerometer provide properly values.
- LDR provide properly values.
- Sensors data is transmitted to the collector.
- LEDs and buzzer work properly.

Then a final test has been developed to verify that the sleep–wake cycle is working properly.

- Sleep and wake by time in every processing cycle.
- Sleep by time and wake by activity (tilt). A 15-min timeout has been configured to sleep if no activity.

**Figure 12:**
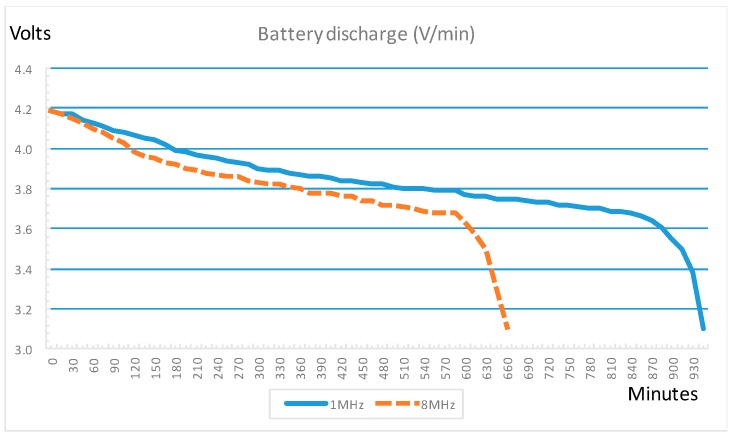
Battery discharge.

Figure 12 shows the discharge process when the cube runs a test program that is continuously doing the following tasks:

(1) Reads the accelerometer sensor and sends the values.
(2) Reads the light sensors and sends the values.
(3) Sleeps during 4 ms.

Discharging battery tests have been made with two different configurations of microcontroller clock, 1 MHz and 8 MHz. The ordinate axis in Figure 12 represents the voltage of the battery and the abscissa axis represents the time in minutes. We can observe that the battery duration in this case is 650 min (more than 10 h and 30 min) for 8 MHz clock and 960 min (16 h) for 1 MHz clock. Thus, using a 1 MHZ clock, the battery duration is 48% higher.

This test simulates an extreme case, but in a real situation the cubes will read sensors data, process them and send results to the collector, only in case of motion or light changes being detected, so the duration will be longer.

**Figure 13:**
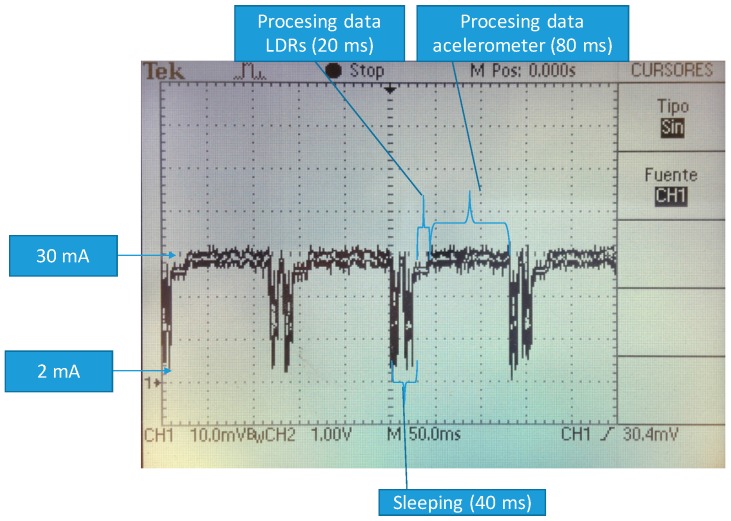
Time variability of energy consumption.

Other tests have been performed without sleeping the microcontroller and the results have been that battery duration is reduced by about 7% for 1 MHz clock. The sleep mode has little effect because the sleeping time (around 40 ms) is less than processing time (around 100 ms) (Figure 13).

The accelerometer process, explained in next section, is critical in time requirement because it must read data from sensor and process them in real time for the purpose of calculating the motion pattern parameters. Thus, a test of the accelerometers process has been performed with the two configurations of clock 1 MHz and 8 MHz. We have concluded that the process must be taken more than 10 times per second in order to obtain valid parameters. This period is only possible using a clock of 8 MHZ, so this is the frequency selected to configure the microcontroller. On the other hand, using this clock frequency, the battery duration is enough to carry out the activities.

### 2.3. Sensing Movement Pattern

These smart cubes are able to capture some data that are not usually available for psychologists when they are working with children and toys. The data they use nowadays are the time that children use to create the tower of cubes, and a qualitative observation about how children move the cube (fast, slow, shaken, etc.). By using the MPU-9150 (InvenSense (Sunnyvale, CA, USA)), we have access to three variable sets: acceleration, gyro angles and magnetometer. The MPU-9150 is a System in Package (SiP) that combines two chips: the MPU-6050 (InvenSense (Sunnyvale, CA, USA)), which contains a three-axis gyroscope; three-axis accelerometer; an on-board Digital Motion Processor™ (DMP™) capable of processing complex MotionFusion algorithms; and the AK8975, a three-axis digital compass. The part’s integrated six-axis MotionFusion algorithms access all internal sensors to gather a full set of sensor data (Description obtained from InvenSense® technical information sheet.).

The MPU-9150 is calibrated with the following parameters:

- Gyro read rate: 1 kHZ.
- Magnetometer read rate: 10 Hz.
- Magnetometer full-scale: ±1200 μT, using a 13 bits ADC.
- Accelerometer read rate: 21 Hz.
- Full-scale accelerometer range: ±2 g, using a 16 bits ADC.
- Gyroscope read rate: 20 Hz.
- Full-scale gyro range: ±250 degrees/s, using a 16 bits ADC.

With these sets of data, we should be able to obtain several interesting values from the actual cubes movement:

- Maximum acceleration.
- Maximum speed.
- Average speed.
- Shaking values.
- Maximum spinning.

Once the basic parameters are read form the MPU-9150, some calculations are made to calculate more elaborated parameters (global acceleration, global average speed, maximum values, etc.), as explained below. Although it is possible to do it faster, experimental results show that a good rate for generating these parameters is about 30 Hz. Some tests show this is consider sufficient to catch the details of the movement.

Of course, the time that the child uses to move the cube from the original position to the end position will be obtained from the Arduino chip itself, and not from the MPU-9150.

**Figure 14:**
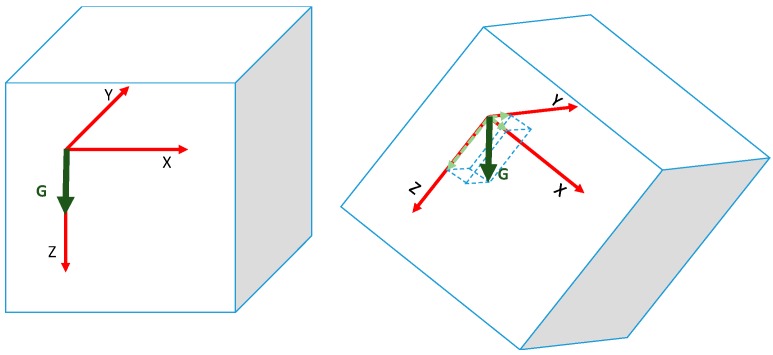
G vector in a stopped and in a spun cube.

Apart from the obvious, most of these values (speed and shaking values) depend on acceleration values. As said before, MUP-9150 device gives the acceleration values on three axes: X, Y and Z. The first problem we have to face is the G value. The acceleration values are not zero when the device is stopped. The G value (9.8 m/s^2^) is always present. If the accelerometer is in a flat position, with the Z-axis perpendicular to the surface, G is a vector that moves from the device to the centre of the earth, so it appears on this axis (with a positive or negative value depending on the axes layout). The problem is that if we move the cube, this value appears on the other axes or in a combination of all of them, as shown in Figure 14. Therefore, the first task is to eliminate this fixed value from the accelerometer.

**Figure 15:**
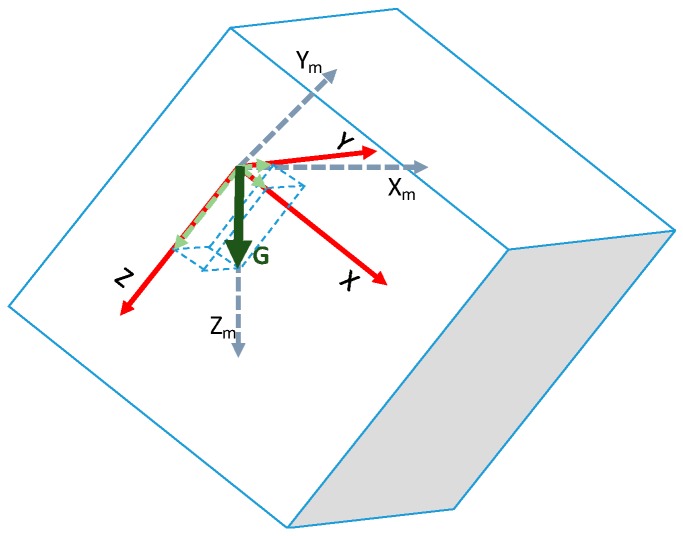
The fixed magnetometer and the acceleration axes.

This is only possible if we know the real position of the accelerometer, and how the X-, Y- and Z-axes are oriented. To obtain this position, we need to use the magnetometer values from the MPU-9150. These devices keep a fixed reference where the axes X_m_ points to the magnetic North, Z_m_ to the centre of the Earth, and Y_m_ is located π/2 rads from the other two (the dashed blue lines in Figure 15).

**Figure 16:**
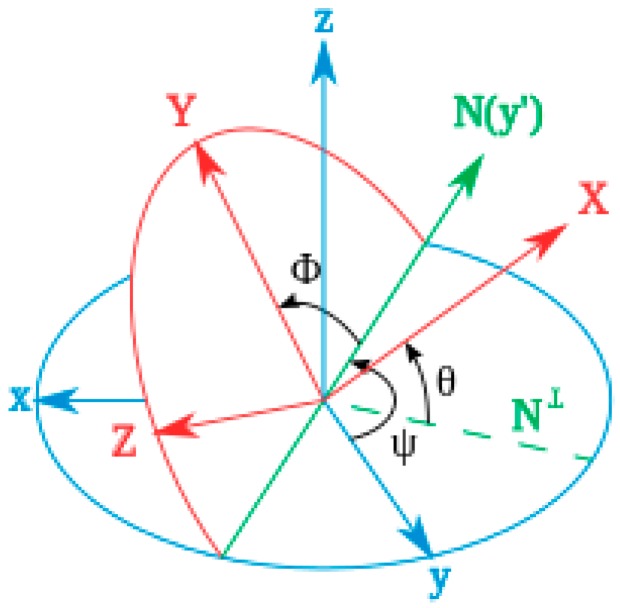
Tait–Bryan angles Yaw (ψ), Pitch (φ), and Roll (θ) from Wikimedia (author: Juansempere).

We may obtain from the MPU-9150 magnetometer the orientation of the X-, Y- and Z-axes of the device through three angle values known as Euler or Tait–Bryan angles: yaw, pitch and roll, as shown in Figure 16.

In the case of the library, we have accessed MPU-9150, which provides Tait–Bryan angles, so we have to take into account that the rotation of the angles is made in ZYX order, as is used in aeronautics [19]. The G vector in the fixed axes XYZ is (0,0,1) (in G—9.8 m/s^2^—units), so the way to take out the effect of G from acceleration values is

*NewAX = AX + sin(φ)*

*NewAY = AY − cos(φ)·sin(θ)*

*NewAZ = AZ − cos(φ)·cos(θ)*

In any case, these kinds of sensors have a noise-bias error that should be taken into account. Even with average acceleration values in the range of [0.005, 0.04] in G units for every axis when the device is completely stopped, we will have problems with the values obtained when speed is calculated, as we will see in the following paragraphs. In order to solve this problem, bias estimation is needed. In our implementation, we consider the acceleration values for every axis when the device is stopped, and we calculate the average of the last N values (10 in the prototype). When the device is moving, we subtract the obtained bias value to every data.

Once we have clean values for acceleration for each one of the three axes, we compute the module of the acceleration vector

$$ModAcc=\sqrt{NewAX^{2}+NewAY^{2}+NewAZ^{2}}$$

This is the value we use to calculate the maximum acceleration that the cube will send to the collector system. In fact, we do not consider the acceleration obtained in each axis as independent values. As the maximum acceleration may be done in any direction, we aggregate the three axes values. Physical therapists consider that the important value is the acceleration module, as it shows the strength of the movement, regardless of its specific direction.

**Figure 17:**
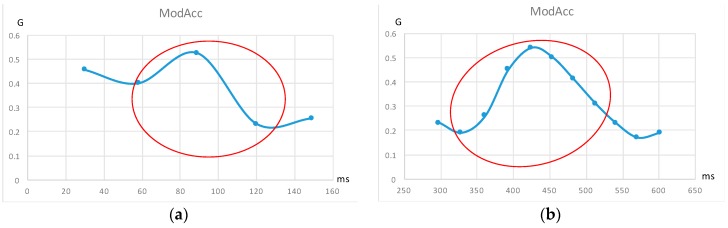
Local maximum of level 1 (**a**); and Local maximum of level 3 (**b**). Vertical units are fractions of *g* (9.8 m/s^2^).

This acceleration value, *ModAcc*, may oscillate, increasing and decreasing, as children shake the cube. In this case, the observer may want to know some measures about the quality of the shaking, mainly due to hand trembling. In order to do this, a shaking vector is computed and sent to the collector system. What do we consider a shake and how do we categorize it? As the module for acceleration vector is always a positive value, every local maximum of this variable will be computed. In order to differentiate hard trembling from soft trembling, we compute a shaking vector by counting every local maxima of levels 1, 2, 3 and 4+. As data are obtained every 10 ms, we count the number of collected data between a local minimum and the following local maximum, and from this local maximum to the following local minimum. The lowest value of these two numbers is the level for that given shake. Figure 17 shows a couple of examples: the first one, on the left, shows a local maximum of level 1. As we can see on the red ellipse, there is only one step from local minimum to local maximum and from this maximum to the next local minimum. The second one, on the right, shows a local maximum of level 3. Note that, in this last case, there are three steps from the local minimum to the local maximum, and five steps from the maximum to the following minimum. In this case, the lowest value is 3, so it is the level of the shaking value. The 2 and 4+ levels are obtained in the same way. In fact, the level 4+ groups all the local maxima for levels 4, 5, 6, etc. This value has been decided after several experiments, and seems to be a good maximum level for practical use. The shaking vector counts the number of level 1, 2, 3 and 4+ shakes made within the analysed movement.

In order to decide if the device is moving or if it is stopped, we use the gyroscope values, which we obtain from the used library in °/s. This sensor is really very sensible to any device manipulation. We fuse the three values GyroX, GyroY and GyroZ (in degrees/s) by using the module of a 3D vector made of these values (MGyro). The physical meaning of this vector is not clear, but the module is useful to know if the cube is being manipulated. When this module is higher than a threshold value (set as 6 in the first prototype, obtained from real experimentation), we consider that the cube is being moved. In fact, if we get a very high value for this variable (typically, more than 200 degrees/s), it may be understood as the child moves the cube spinning it, while lower values (below 50 degrees/s) mean that the cube is moved in a straight path. In this sense, the maximum value of a movement may give some valuable information to educators when they analyse the data.

Another problem comes from spurious movements. These may come from table movements, small touches, etc. To avoid these false data, we only consider real movement when it happens, at least, for a given threshold value (set as 0.2 s in the prototype).

Once we have the tools to decide when the cube is moving, and the unbiased acceleration values, we can afford the speed values. As the reader knows, the speed may be obtained integrating the acceleration values. As we have three acceleration components, we have to integrate each one of them to obtain three speed components, V_X_, V_Y_ and V_Z_. At this moment is when the bias value for acceleration is really important. To get the speed, an integration is done to obtain the area of the acceleration values from the starting movement time. Now, any bias value on it will accumulate providing an increasing value for speed. For instance, if the acceleration value for the X-axis has just a bias of 0.01 G (0.098 m/s^2^), the speed value will rise in 10 s from 0 to 0.98 m/s, even with the device completely stopped. If we subtract the estimated bias for all the three axes, this error will be reduced to acceptable values (considering that children will be moving the cubes only for a few seconds). Once we get the speed components, we can calculate the module of the speed vector, and compute the maximum as well as the average speed for the cube movement (from the starting movement to the stop time).

Of course, when the child has to build a cube tower, the collector will store data from the different cube movements. For every child, a set of vectors will be used to create a “personal pattern”. The nine variables that form these vectors are Time to move the cube, maximum acceleration, the four values of the shaking vector, maximum speed, average speed and maximum spinning. Taking into account that, at this moment, educators only consider Time to move the cube and a rough qualitative value for shaking, we assume that these values should be useful to detect, in a more accurate way, developmental problems in children.

### 2.4. Sensing Hide Face

An important part of each experiment is to determine the relative position of a cube from the other cubes. In fact, the data obtained from movement from MPU-9150 do not allow obtaining the position of the cubes, so the only way to determine the position of the different cubes is to use other sensors. To perform this task, the cube will use Light-Dependent Resistor (LDR). This component varies its resistance depending on the intensity of light received. In our specific sensor, the resistance value is around 400 Ohm when there is a light intensity of 1000 Lux, and for a light intensity of 10 Lux, the resistance changes to approximately 9 KOhm. The intermediate values follow a logarithmic curve, so a value of 1 KOmh is used as threshold between light and darkness. These sensors give information about the relative position of two cubes because if they are near enough, the LDR does not receive light.

The fusion of the data provided by these sensors from each cube allows understanding the evolution of the cubes layout while the experiment is performed.

By using one centred LDR in every cube face, it is possible to decide which faces are covered and which other are uncovered. In order to increase the information provided to the experts, we have designed a system based on a couple of LDRs in every face. These LDRs are located at the corners of one of the two diagonals of the face. With this layout the cubes may offer information about the quality of contact between cubes. If both faces are aligned, both LDRs will be covered. If only one LDR is covered, the cube will give information letting know the experts that those cubes are not in row.

While this idea is perfectly possible to implement in a conventional Arduino board, due to the small size of the whole system, the microcontroller that has been used leaves only six available analogue inputs. In this case, we have had to make an electronic design in which two LDR sensors are joined as may be seen in Figure 9.

In this design, the analogue inputs take the reference voltage (V_ref_) of their respective sensors. In order to obtain a more reliable measure, each sensor connected to the same V_ref_ will be placed on different sides of the cube. This will allow us to avoid cases of uncertainty when deciding if a face is covered or not while preventing possible hardware failures. If we placed both sensors in the same side of the cube, the V_ref_ measures in that face will be the same whether the face is covered or not.

The following considerations need to be taken into account to explain the distribution of sensors in the cube surface:

- The six V_ref_ measures are identified with characters (*a, b, c, d, e* and *f*). In this case, each V_ref_ corresponds with the microcontroller pinout A0, A1, A2, A3, A6 and A7, respectively.
- In each side of the cube, one sensor is always connected to V_cc_ and the other one is connected to GND.
- Sensors that provide the same measure will be always placed in different sides of the cube.

**Figure 18:**
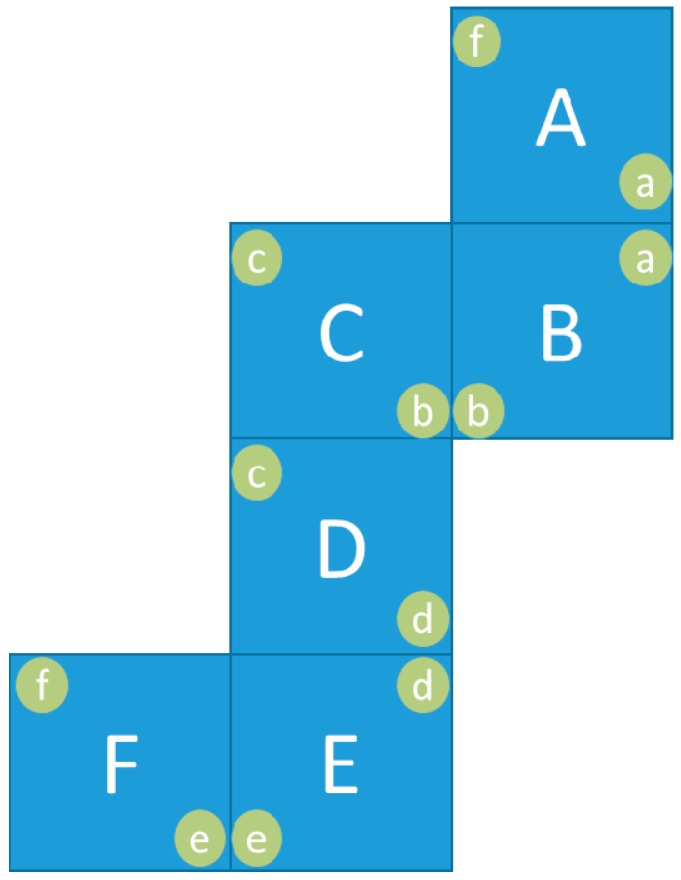
Sides and LDR location with names.

Therefore, as it is shown in Figure 18, the sensors are connected in pairs (*af, ba, cb, dc, ed* and *fe*). The first character of the pair identifies the LDR connected to GND and he second one identifies the sensor connected to V_cc_ (See the schematic of LDRs in Figure 9).

Additionally, one of the main requirements of the system is to reduce the amount of data sent from the cubes to the collector. Therefore, we have developed a codification of the measurements that transform them into a unique integer value identifying the faces that are covered in each case.

The codification is based on grouping the voltage values received from the sensor in three different levels. Level 1 will include the read values that are characterized as low, level 2 will include high voltage values, and level 0 will include the range of values between high and low. It is important to note that it is possible to receive an intermediate value (level 0) when reading the pair of sensors both when the face is uncovered or covered.

Each value read is processed as a ternary base. The level value read in each side of the cube is codified with a different weight in the ternary base as it is shown in Table 3. The combination of the values and weighs determines the unique integer that will be transmitted.

The header (shaded cells) of the Table 3 represents the covered faces. The values of each column are the levels obtained from the voltage values in each pair of sensors. For instance, when a cube has the face “A” covered, the collector will receive a “7” value (1 × 1 + 3 × 2). If the face covered is “E”, the sent value will be 567 (81 × 1 + 243 × 2). As has been pointed before, these values are the result of the sum of level values in the column multiplied by the values of the weight column.

Table 4 represents another example of the integer values calculated to identify the different combinations of three covered faces in a cube. In this case, there are 0 level values displayed. This is because of the possibility of uncertainty in the values received from the sensors, as we cannot know if the sensors are active or not. We do not want to include this uncertainty in the obtained value, and, therefore, we add a 0 value.

In the following array, we can see the integer values obtained for each combination of covered faces in a cube using the codification explained before:

Values = ((7, “A”), (21, “B”), (63, “C”), (189, “D”), (567, “E”), (245, “F”), (19, “AB”), (70, “AC”), (196, “AD”), (574, “AE”), (249, “AF”), (57, “BC”), (210, “BD”), (588, “BE”), (266, “BF”), (171, “CD”), (630, “CE”), (308, “CF”), (513, “DE”), (434, “DF”), (83, “EF”), (55, “ABC”), (208, “ABD”), (586, “ABE”), (261, “ABF”), (178, “ACD”), (637, “ACE”), (312, “ACF”), (520, “ADE”), (438, “ADF”), (87, “AEF”), (165, “BCD”), (624, “BCE”), (302, “BCF”), (534, “BDE”), (455, “BDF”), (104, “BEF”), (495, “CDE”), (416, “CDF”), (146, “CEF”), (29, “DEF”), (163, “ABCD”), (622, “ABCE”), (297, “ABCF”), (532, “ABDE”), (450, “ABDF”), (99, “ABEF”), (502, “ACDE”), (420, “ACDF”), (150, “ACEF”), (33, “ADEF”), (489, “BCDE”), (410, “BCDF”), (140, “BCEF”), (50, “BDEF”), (11, “CDEF”), (487, “ABCDE”), (405, “ABCDF”), (135, “ABCEF”), (45, “ABDEF”), (15, “ACDEF”), (5, “BCDEF”), (0, “ABCDEF”))

The integers obtained are unique for each combination of covered faces, so it is a simple task to decode the identifiers of covered faces from the number by mapping values and cases. For instance, if we have the value 208 (we can see it in the last value list and in Table 3), this is equivalent to having the faces “A”, “B” and “D” covered.

The information about the faces of the cubes is sent to the collector each time the value changes (and consequently, the status of the faces of the cube have changed). This information will be analysed a posteriori to determine the figures (e.g., the tower shown in Figure 1) created with the combination of cubes during an experiment.

### 2.5. Collector System

As shown in Section 2, the physical architecture of the system is composed of a series of smart toys that are going to connect themselves to a data collector. This collector is able to store the information received from the sensors embedded in each toy and is managed through client applications from tablets or laptops.

Physically, the collector is deployed inside a Raspberry Pi board. The Raspberry is provided with a Radio Frequency device (NRF24L01+, the same as that used in the cubes) for toys–collector communications, and a USB WiFi adapter (Realtek RTL8188CUS) for client–collector communications. It also includes a RFID reader (RFID-RC522) for the optional automatic identification of users (both professionals doing experiments and children using the toys). The Raspberry Pi runs Raspbian Wheezy as the operating system.

**Figure 19:**
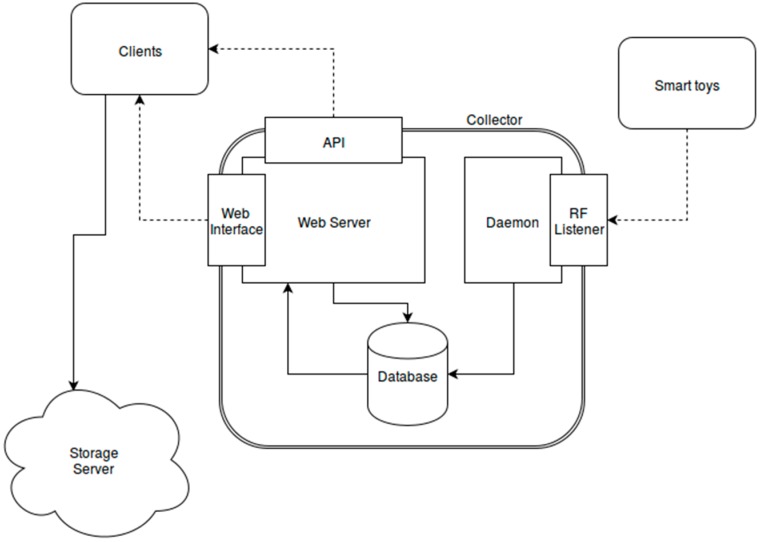
Collector Software architecture.

**Figure 20:**
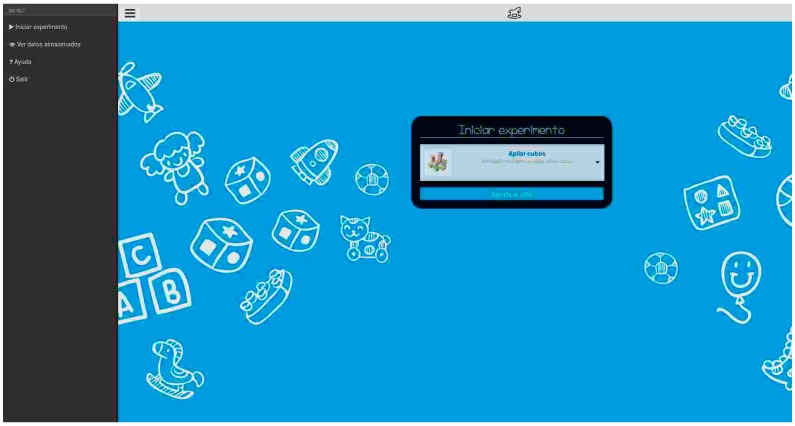
Screenshot of the collector web interface.

The collector software architecture is shown in Figure 19, and is composed of three main components:

1. **A Web Server**: The server is the main software component of the collector. It is developed using Python and the Django Framework, and provides the main functionalities for professionals to interact with the collector. The main functionalities offered are:
  - Provides a web interface to interact with the collector using a web client. In Figure 20, a screen capture of the developed web interface is shown.
  - Provides an API to interact with the collector using client applications.
  - Allows professionals to start and stop experiments.
  - Allows professionals to obtain data files stored from experiments.
2. **A system daemon**: This daemon is used to receive data from the sensors. It uses the NRF24 library to open a communication channel through the RF device and waits for the data from the cubes to be received. It stores the data temporarily in a database. It is controlled through the web server, and will be started and stopped for each experiment.
3. **A database**: The database is used for two main purposes. It is used as a temporary storage for data from experiments (until they are stored in encrypted files when the experiment is finished) and is also used as a management database for auxiliary data used by the web server (child and professional identifiers, activity information, etc.)

**Figure 21:**
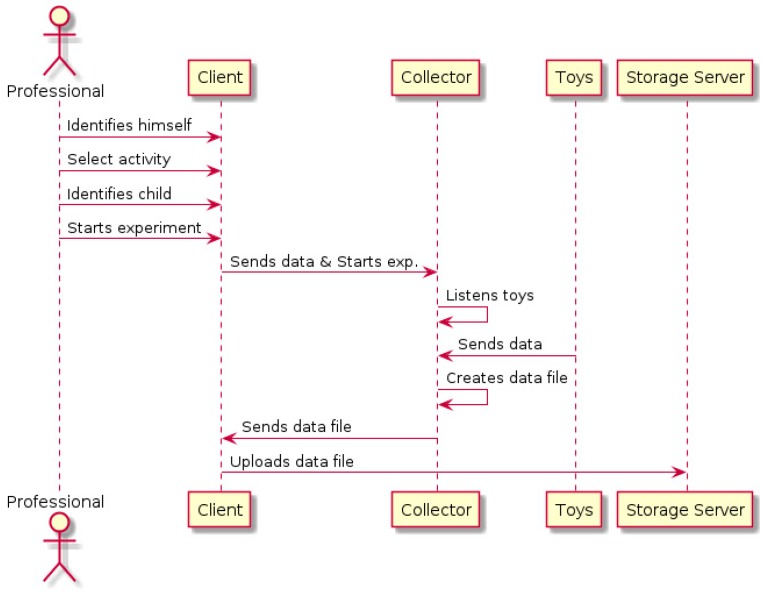
Data recollection process.

The process of data collection is described in Figure 21. The process starts when a professional identifies himself in the system (using the web interface, through an application that queries the API or using is RFID card directly in the collector reader). He can then choose an activity to perform and identify the child that will play (to allow future historical analysis of all the experiments performed with the same child). When the professional starts the experiment, the system daemon will be executed and the collector will start waiting for data from the toys. The professional can stop the experiment at any moment using the client interface.

Both the professional identification and the children identification are based in numeric identifiers, and can be performed introducing manually the identifier using the client interface or using the RFID reader of the collector (given that the professional has been provided with RFID cards to identify himself and the children). Not using any personal data to identify users is intended to provide privacy, as it is a very important topic in this system, especially for children involved in the experiments. Using this identification system allows storing data in the collector without the risk that someone has access to them and can deduce the real identity of the people involved in the experiments.

The mapping between these identifiers and personal data will only performed in a storage server that provides enough security to assure that only authorized users can access to the data.

Once the experiment is finished, the data received in the collector will be stored in a plain text file, including some extra data about the experiment (professional and child identifiers, start and stop timestamps, etc.). This file will use JSON format to store data. The following is an example of the generated data:
{ “ID_experiment”: “2015-09-28-15-21-34.154680”, “ID_activity”: “2.12”, “ID_child”: “244119”, “Start”: “2015-09-28-15-21-34.154680”, “Stop”: “2015-09-28-15-21-45.353661”, “Data”: [ … ],}

The data included in the file will be composed of JSON structures that provide information about how the toys behave during the experiment. As has been explained in previous sections, each cube will provide information about its movement and the faces that are covered or uncovered. The following is an example of generated data:
{ “timestamp”: “2015-11-18 09:08:30.251626+00:00”, “sensor_id”: “1”, “data”: { “max_accel”: “0.624094903469”, “max_speed”: “0.294163644314”, “activity_time”: “3572”, “avg_speed”: “0.186813503504”, “max_mov”: “347.157592773” “shaking1”: “6”, “shaking2”: “1”, “shaking3”: “0”, “shaking4”: “0”, }, “device_id”: “5”},

Due to the sensitive nature of the data collected (although no professional or child personal data are stored as explained before), some security considerations must be addressed, in order to assure that only the authorized users can access to it. To achieve this, the data files generated in the collector are automatically encrypted before they can be obtained by users. We use a symmetric encryption system called Fernet (Fernet is part of the Python Cryptography library: http://cryptography.readthedocs.org/en/latest/fernet/) that is built on top of AES on CBC mode with a 128-bits key. It also uses HMAC with SHA256 for authentication. A new key used is generated randomly for each experiment and is stored in the same file using a RSA public key to encrypt it. The corresponding private key is stored securely in the storage server, so this is the only system that can decrypt the data.

The files generated in the collector can be obtained through the web interface, the API, or via an USB device connected directly to the collector. This latter way or file retrieval has been included to be used in environments where the client used to interact with the collector has no storage capacity. The collector will detect the USB device connected to it and will copy the selected encrypted file to it.

## 3. Conclusions

When we are working in the field of early detection of developmental delay in children, information is something precious. In this area of study, the use of smart toys, such as the cube presented here, might radically change the way in which experts (pedagogues, psychologists, physiotherapists, etc.) work.

Although the nature/core/procedure of the experiments in which the toy is now used has not changed, new results could be obtained. Earlier findings will probably be found but with more precision, as well as new data that were not possible to be obtained before.

Besides the classic data, these cubes will give additional information, e.g., quality of the figure of performance, or “shaking quality” of the cube’s movement, from the starting position to the destination.

All these additional data enable the detection of possible developmental motor difficulties or disorders. By having parameterized data, if the experiments are repeated, you may see the evolution of various children and the comparison among them, an interesting possibility in the study of Developmental Coordination Disorder (DCD) [20].

The centralized collector-based architecture developed will allow an easy but powerful and flexible interaction with the cubes. This scheme allows non-technical users to perform experiments using the cubes and obtain meaningful data without interacting directly with the sensors. The architecture has also been designed to be easily extended with future new toys or experiments that can be performed using the interfaces provided, by just including the new activities as an option in the collector server.

## Figures and Tables

**Table 1:** Taxonomy of games.

Area of Health Activity	Personal	Professional Practice	Research and Academia	Public Health
Preventative	“Exergaming” Stress	Patient communication	Data collection	Public-health messaging
Therapeutic	“Rehabitainment” Disease management	Pain distraction Cyberpsychology Disease management	Virtual humans	First responders
Assessment	Self-ranking	Measurement	Inducement	Interface and visualization
Educational	First aid Medical information	Skills and training	Recruitment	Management simulations
Informatics	Personal health records	Electronic medical records	Visualization	Epidemiology

**Table 2:** Communication technologies comparative.

Device	Size	Consumption
NRF24L01+ mini	12 × 18 mm^2^	15 mA
Bluetooth HC-05	28 × 15 mm^2^	50 mA
WIFI 8266 ESP-03	12 × 17 mm^2^	70 mA

**Table 3:** Values with a single side covered.

	Sensors	Weight	A	B	C	D	E	F
Side A	af	1	1					2
Side B	ba	3	2	1				
Side C	cb	9		2	1			
Side D	dc	27			2	1		
Side E	ed	81				2	1	
Side F	fe	243					2	1
	Value		7	21	63	189	567	245

**Table 4:** Three covered sides (“A”, “B” and other).

	Sensors	Weight	ABC	ABD	ABE	ABF
Side A	af	1	1	1	1	0
Side B	ba	3	0	0	0	0
Side C	cb	9	0	2	2	2
Side D	dc	27	2	1		
Side E	ed	81		2	1	
Side F	fe	243			2	1
	Value		55	208	586	261
